# Correction: Single-cell RNA-seq reveals transcriptomic heterogeneity mediated by host–pathogen dynamics in lymphoblastoid cell lines

**DOI:** 10.7554/eLife.75422

**Published:** 2021-11-11

**Authors:** Elliott D SoRelle, Joanne Dai, Emmanuela N Bonglack, Emma M Heckenberg, Jeffrey Y Zhou, Stephanie N Giamberardino, Jeffrey A Bailey, Simon G Gregory, Cliburn Chan, Micah A Luftig

**Keywords:** Human, Viruses

2021. SoRelle ED, Dai J, Bonglack EN, Heckenberg EM, Zhou JY, Giamberardino SN, Bailey JA, Gregory SG, Chan C, Luftig MA. Single-cell RNA-seq reveals transcriptomic heterogeneity mediated by host–pathogen dynamics in lymphoblastoid cell lines. *eLife* **10**:e62586. doi: 10.7554/eLife.62586

Published 27 January 2021

After publication, it came to our attention that the M81 strain of EBV used in this work was incorrectly referred to as a Type 2 EBV strain within the Key Resources table. While the M81 strain contains the V3 version of the Z promoter and is thus spontaneously lytic due to higher Z expression (a characteristic of Type 2 EBV), it is a Type one strain. We have corrected the manuscript as follows to resolve this error:

#1

Original text:

[ Key resources table, row 3, “Additional Information” column ]

Stimulated to obtain M81 strain (Type 2 EBV) viral supernatants

Corrected text:

[ Key resources table, row 3, “Additional Information” column ]

Stimulated to obtain M81 strain (Type 1 EBV) viral supernatants

#2

Original text:

[ Within the Author response section ]

The contributions from testing different viral strains (B95-8 and M81, the most widely used Type one and Type two strains, respectively) in cells from the same donor are articulated throughout the manuscript.

Corrected text:

[ Within the Author response section ]

The contributions from testing different viral strains (B95-8 and M81) in cells from the same donor are articulated throughout the manuscript.

The article has been corrected accordingly.

